# Preparation and Characterization of Superabsorbent Polymers Based on Sawdust

**DOI:** 10.3390/polym11111891

**Published:** 2019-11-15

**Authors:** Mingchang Zhang, Shaodi Zhang, Zhuoran Chen, Mingzhi Wang, Jinzhen Cao, Ruoshui Wang

**Affiliations:** 1 MOE Key Laboratory of Wooden Material Science and Application, Beijing Forestry University, Beijing 100083, China; zhangmingchang0717@163.com (M.Z.); zhang384@bjfu.edu.cn (S.Z.); chenzhuoranran@163.com (Z.C.); caoj@bjfu.edu.cn (J.C.); 2 School of Soil and Water Conservation, Beijing Forestry University, Beijing 100083, China; wrsily_2002@163.com

**Keywords:** sawdust, polymerization, superabsorbent polymer, reusability, water retention agent

## Abstract

Sawdust, a cheap by-product of the timber and forestry industry, was considered as a framework structure to prepare superabsorbent polymer with acrylic acid (AA) and acrylamide (AM), the synthetic monomers. The effects of initiator content, crosslinker content, AA content, AM content, degree of neutralization of AA, and reaction temperature on the swelling rate of superabsorbent polymer were investigated. The synthesized polymer was characterized by Fourier transform infrared spectroscopy (FTIR), X-ray diffraction (XRD), scanning electron microscopy (SEM), and thermogravimetric analysis (TGA). Under optimal synthesis conditions, the results showed that the swelling rate of the polymer in deionized water and 0.9% NaCl solution reached 738.12 and 90.18 g/g, respectively. The polymer exhibits excellent swelling ability, thermal stability, and reusability. After the polymer was introduced into the samples (soil or coal), the water evaporation rate of the samples was significantly reduced, and the saturated water holding capacity and pore structure were also significantly improved.

## 1. Introduction

Superabsorbent polymer (SAP) is a new material with a three-dimensional network structure [1,2,3]. It can absorb as much water as hundreds to thousands of times of its own weight in a short time without dissolving [4,5,6]. Currently, SAP has been widely used in medical [7], agricultural [8,9], commodity [10], environmental pollution treatment [11,12], and other fields [13,14]. However, more than 90% of SAPs are non-recyclable products, most of which are synthesized from petroleum-based monomers, causing serious environmental pollution [15,16]. Consequently, the synthesis of SAP based on natural polyhydroxy materials has become a hot research issue [17,18].

In recent years, bio-waste, especially agricultural by-products such as starch [19], soy protein [20], and straw [21,22,23], have been considered for the preparation of SAP. These bio-wastes are non-toxic, renewable, biocompatible, and biodegradable. The proposal of this scheme not only improves the utilization efficiency of biological waste, but also reduces the production cost of SAP. Nevertheless, such materials usually require chemical reagents for pretreatment before preparing SAP [24,25,26,27]. The cumbersome operating procedures increase the cost of production of SAP products, and the large consumption of chemical reagents leads to serious environmental pollution.

As a by-product of wood machining, the annual output of sawdust in China is up to 24.15 million cubic meters [28]. The early disposal of sawdust is mainly incineration and landfill, which leads to a series of environmental problems. Because of this, the recycling and reuse of sawdust has drawn much attention in recent years.

Graft polymerization is a common method of chemical modification, which can achieve the expansion and improvement of sawdust performance. These grafting methods include electron beam irradiation [29], microwave irradiation [30], plasma [31], ozone [32], photometry [33], and chemical methods [34]. The solution polymerization, a kind of chemical grafting method, has the advantages of easy mixing and heat transfer, uniform catalyst dispersion, easy temperature control, and avoiding local overheating. The active groups of sawdust, such as hydroxyl and carboxyl groups, become active sites in the presence of free radicals, where the synthetic monomers are grafted onto the sawdust. Finally, graft copolymer is prepared under crosslinking of the crosslinker. There have been many reports on the graft of synthetic monomers onto the surface or into the interior of the wood. Wang et al. [35] grafted octadecyl isocyanate onto the wood cell wall, giving the wood surface high hydrophobicity and excellent durability against chemical corrosion. Qiu et al. [36] inserted water-soluble vinyl monomer into the wood cell wall for graft polymerization to improve the mechanical properties of wood. Cabane et al. [37] used wood as a large porous frame template and assembled a styrene monomer onto the wood cell wall to prepare functional wood with superhydrophobic properties. As a result, sawdust can react with monomers sufficiently and be the candidate for preparing SAP as a copolymer and structural component. Furthermore, many studies on crosslinking graft copolymerization of acrylic acid and acrylamide monomers onto biological materials or its derivatives have also been reported [15,38]. Based on these findings, we propose to use raw sawdust to synthesize SAP, rather than the modified products obtained after chemical treatment of biomaterials. This design simplifies the cumbersome process and limits the use of chemical reagents during the SAP preparation. Moreover, it is beneficial for efficient utilization of sawdust and for reducing the preparation cost of SAP.

In this study, by one-step polymerization, several groups of sawdust-graft-acrylic acid-graft-acrylamide (SW-AA-AM) polymers were synthesized and characterized by FTIR, XRD, SEM, and TGA. Subsequently, the influence of vital factors on the swelling properties of the SW-AA-AM polymer was investigated. The water retention properties, thermal stability, and reusability of the SW-AA-AM polymer were also analyzed. Finally, the performance of the SW-AA-AM polymer was studied as a water retention agent for soil or coal.

## 2. Materials and Methods

### 2.1. Materials

The reagents used in this work including acrylic acid (AA), acrylamide (AM), sodium hydroxide (NaOH), ammonium persulfate (APS), and *N,N’*-methylenebisacrylamide (MBA) were all analytical reagent grade. They were purchased from Maclean Biochemical Technology Co., Ltd. (Shanghai, China). Sawdust is derived from fast-growing fir (*Cunninghamia lanceolata*) and was provided by Huzhou Forest Farm (Huzhou, China). The soil used in this study was collected from Beijing’s umber, and the coal powder was purchased from the soft coal of Wuhai Coal Mine (Wuhai, China). All the particles were passed through a 60-mesh screen.

### 2.2. Preparation of SW-AA-AM Polymer

First, 1 g of dry fir sawdust was placed in a 200 mL four-necked flask equipped with a thermometer, a stirrer, a reflux condenser, and a nitrogen tube; 50 mL of distilled water was then added. The mixture was stirred in a water bath under different temperatures. Nitrogen flux was injected after the system reached the specific temperature to remove oxygen from the aqueous solution. Subsequently, APS was dissolved in the solution in the flask carefully. Fifteen minutes later, neutralized AA (neutralized AA with different neutralization degrees were prepared by adding AA to NaOH solution under ice bath conditions and used immediately) [39], AM, and MBA were added to the flask in sequence and reacted at a constant temperature for 3 h to obtain a crude product. In order to remove the unreacted reagent and soluble polymer, the crude products were dispersed in a beaker containing anhydrous ethanol for 3 h, and they were taken out and cut into small pieces. These pieces were followed by placing in fresh anhydrous ethanol for another 24 h to get the SW-AA-AM polymer. Finally, the polymer was dried to a constant weight in an oven at 60 °C and ground into powder (all the powders were passed through a 60-mesh screen).

### 2.3. Characterization

#### 2.3.1. FTIR Analysis

FTIR measurements of sawdust and SW-AA-AM polymer powders were conducted by a Vertex 70 Fourier transform infrared spectroscopy (Bruker Company, Karlsruhe, Germany) with potassium bromide pressed-disk technique. The wavelength range was 500–4000cm^−1^.

#### 2.3.2. XRD Analysis

The X-ray diffraction measurements of sawdust and SW-AA-AM polymer powders were carried out on an X’Pert PRO MPD type X-ray diffractometer (Nalytical Company, Almelo, Netherlands) with Cu K_α_ radiation, the data were recorded in the 2θ range of 10°–60° with a scanning speed of 5°/min.

#### 2.3.3. SEM Analysis

The morphologies of sawdust and SW-AA-AM polymer were observed by a JSM-7800 Prime scanning electron microscope (JEOL, Tokyo, Japan). The sawdust and polymer were coated with an Au layer prior to the observation.

#### 2.3.4. Thermal Stability Analysis

The thermal stability of the SW-AA-AM polymer was analyzed using a TGA-Q50 thermogravimetric analyzer (TA Instruments, New Castle DE, Delaware, USA) at a heating rate of 10 °C/min under nitrogen atmosphere. The temperature range was 30–600 °C.

### 2.4. Swelling Performance

A total of 0.5 g of dry SW-AA-AM polymer powder was immersed in 500 mL of deionized water or 100 mL of 0.9% NaCl solution at room temperature for 6 h to reach the swelling equilibrium. The excess water or 0.9% NaCl solution was then filtrated through a 120-mesh filter screen until water or solution ceased to drip. The swelling rate (*SR*) of the SW-AA-AM polymer was calculated by the following formula (1):

(1) $$SR=\frac{m_{2}-m_{1}}{m_{1}}\times 100\%$$

where *m*_1_ (g) is the weight of the dry SW-AA-AM polymer; and *m*_2_ (g) is the fully swollen SW-AA-AM polymer. The swelling rate of the SW-AA-AM polymer in deionized water and 0.9% NaCl solution was SR and SR’, respectively. All data were the averages of triplicate replicates.

### 2.5. Reusability

The SW-AA-AM polymer was first fully swollen in deionized water and then dried in an oven at 60 °C to a constant weight. The above “swelling–drying” process was repeated 5 times and the swelling rate of each time was recorded. The recycling utilization rate (*R*) represents the n times of swelling rate to the initial value, which can be used as an evaluation index of the reusability of the SW-AA-AM polymer [23].

### 2.6. Water Retention Capacity

A total of 15 g of the fully swollen SW-AA-AM polymer was placed in a glass petri dish and dried in an oven at different temperatures; the weight of the SW-AA-AM polymer samples was recorded per hour.

### 2.7. Water Retention Performance in Soil or Coal

The soil or coal was placed in an oven at 80 °C to remove moisture and volatile compounds. A total of 20 g of soil or coal and a certain amount of SW-AA-AM polymer (0, 0.1, 0.2, 0.3, and 0.4 wt %) were blended in a beaker, and 30 g of deionized water was mixed. The mixture was then dried in an oven at 35 °C until the weight reached was constant; the weight of samples was recorded per h. The moisture content (MC) of the samples was calculated by the formula (2):

(2) $$MC=\frac{w_{i}-(w_{1}+w_{2})}{w_{1}}\times 100\%$$

where *w_i_* (g) is the mass of the beaker and the mixture after *i* hours; *w*_1_ (g) is the total mass of the polymer and the soil; and *w*_2_ (g) is the mass of the beaker. All data were the averages of triplicate replicates.

## 3. Results and Discussion

### 3.1. Characterization

#### 3.1.1. FTIR Analysis

**Figure 1:**
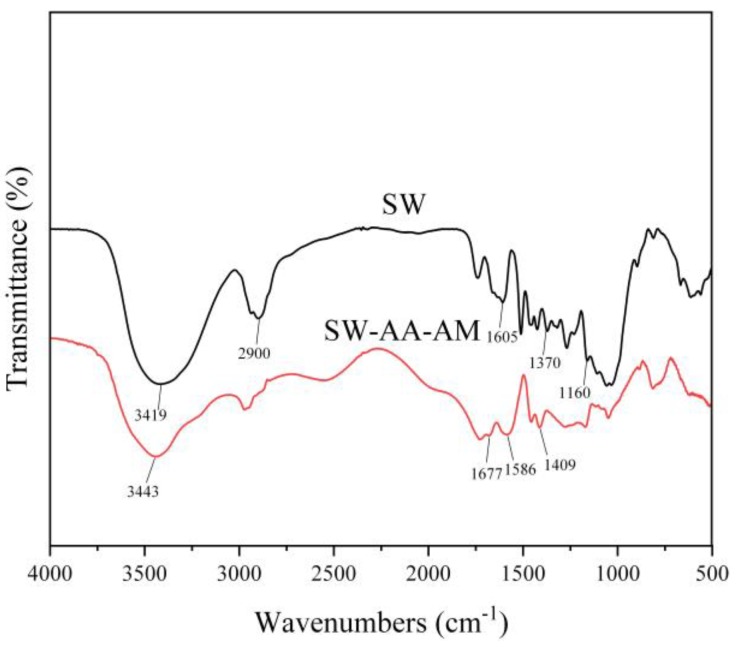
FTIR spectra of sawdust and the sawdust-graft-acrylic acid-graft-acrylamide (SW-AA-AM) polymer.

Figure 1 shows the FTIR spectra of sawdust and the SW-AA-AM polymer. In the infrared spectrum of sawdust, the peak stretching vibration of –OH was observed at 3419 cm^−1^. The band located at 2900 cm^−1^ was assigned to the C–H stretching vibration of the methylene group in cellulose [40,41]. The peak at 1160 cm^−1^ belonged to the stretching vibration of C–O–C in cellulose and hemicellulose. A bending vibrational peak of C–H bonds in cellulose and hemicellulose molecules was observed at 1370 cm^−1^. The peak at 1605 cm^−1^ was attributed to the vibration of the benzene ring carbon skeleton in lignin [42]. This indicates that the sawdust contains cellulose, hemicellulose, and lignin. In the infrared spectrum of the SW-AA-AM polymer, the peaks at 3443 and 1677 cm^−1^ were caused by the stretching vibration of –NH in acrylamide and the stretching vibration of the amide group (–CONH_2_), respectively [39,43]. The new peak at 1409 cm^−1^ was assigned to the stretching vibration of C–N in acrylamide. The new peak at 1586 cm^−1^ was originated from the anti-symmetric stretching vibration of the –COO– group in acrylic acid [44]. In addition, the stretching vibration peak at 1620 cm^−1^ derived from C=C in acrylic acid disappeared. These changes indicate that the SW-AA-AM polymer was successfully synthesized.

#### 3.1.2. XRD Analysis

**Figure 2:**
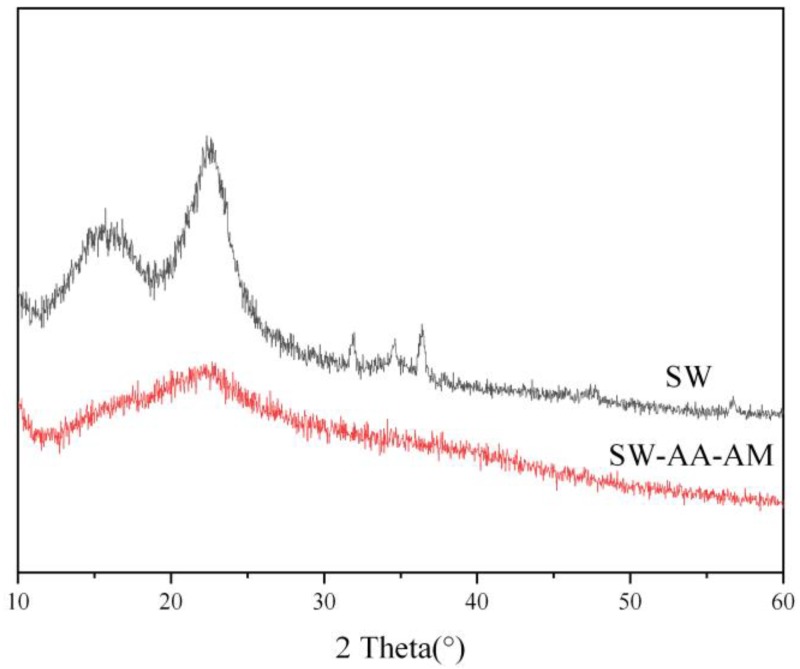
XRD patterns of sawdust and the SW-AA-AM polymer.

In the wood cell wall, cellulose is present in an ordered microfibril bundle state formed by aggregation of molecular chains, and hemicellulose, as well as lignin in an amorphous state penetrating into cellulose [45]. The X-ray diffraction patterns of sawdust and the SW-AA-AM polymer are shown in Figure 2. The XRD pattern of sawdust has two distinct diffraction peaks around 2θ = 16° (amorphous region cellulose II) and 22° (crystalline region, cellulose I), which means that it is a polycrystalline structural material [46,47]. The SW-AA-AM polymer forms a lower intensity diffraction peak in a wider range, indicating that the original polycrystalline structure of the sawdust has been destroyed. This is attributed to the fact that the grafting reaction between the sawdust and the monomer changes the aggregated morphology of cellulose inside the cell wall.

#### 3.1.3. SEM Analysis

**Figure 3:**
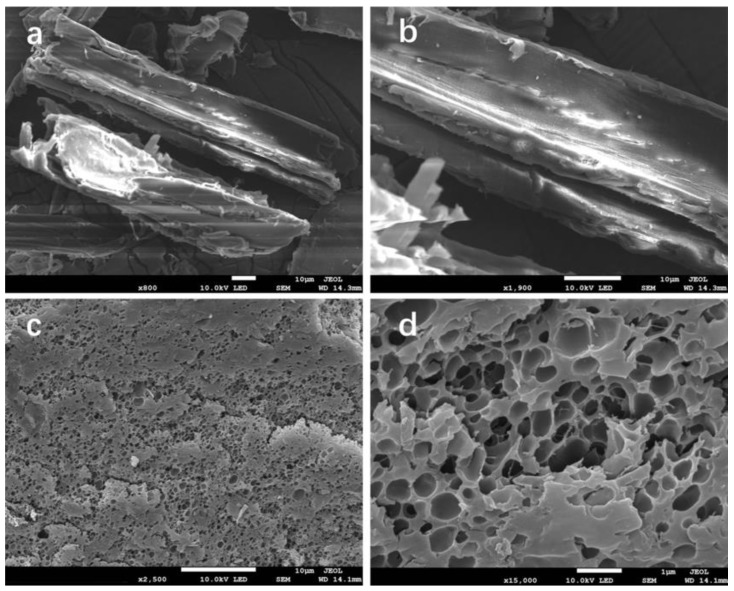
SEM images of sawdust (**a**,**b**) and the SW-AA-AM polymer (**c**,**d**).

The SEM images of sawdust and the SW-AA-AM polymer are shown in Figure 3. The surface of the sawdust is relatively smooth (Figure 3a,b). Compared with sawdust, a large number of continuous microporous structures can be observed on the surface of the SW-AA-AM polymer (Figure 3c,d), consistent with previous reports [21,40]. This structure allows the SW-AA-AM polymer to have a larger specific surface area, which not only facilitates the rapid entry of liquid, but also increases the water capacity of the polymer [48]. The results show that the graft copolymerization reaction changes the original structure of the sawdust.

#### 3.1.4. Thermal Stability Analysis

**Figure 4:**
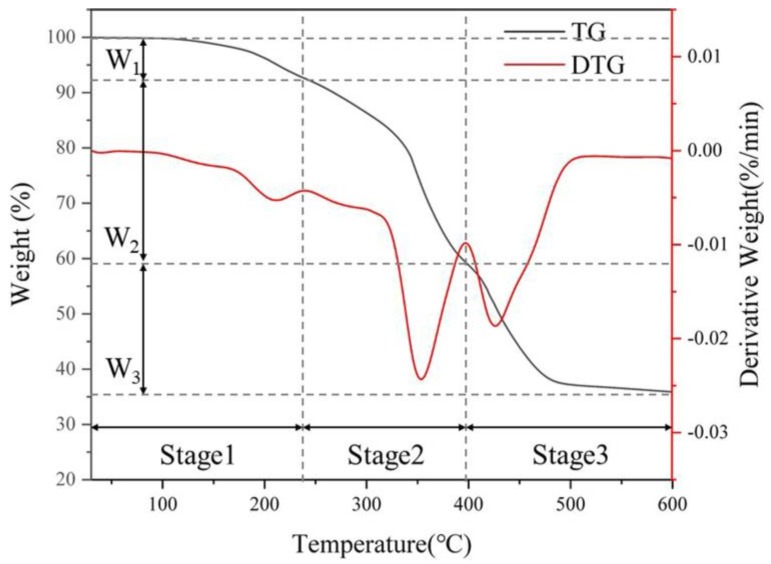
Thermogravimetry (TG) and Derivative thermogravimetry (DTG) analysis of SW-AA-AM polymer (W1, the mass loss of the polymer in the first stage; W2, the mass loss of the polymer in the second stage; W3, the mass loss of the polymer in the third stage).

Figure 4 shows the TG and DTG curves of the SW-AA-AM polymer. As can be seen from the figure, the TG curve is mainly divided into three stages. The mass loss of the polymer in the first stage (30–240 °C) is 8%, mainly due to the evaporation of residual water in the polymer network and the dehydration of acrylic acid to form an acid anhydride [49]. The mass loss of the polymer in the second stage (241–397 °C) is corresponding to the decomposition and oxidation of the carbohydrate chain, and the C–O–C glycosidic bond cleavage of the cellulose chain [40]. The temperature range of the third stage is 398–600 °C, and the mass loss in this stage is caused by the fracture and decomposition of the polymer backbone. This indicates that the SW-AA-AM polymer has good thermal stability and can be used safely even in harsh environments.

### 3.2. Mechanism of SW-AA-AM Polymer Production

**Figure 5:**
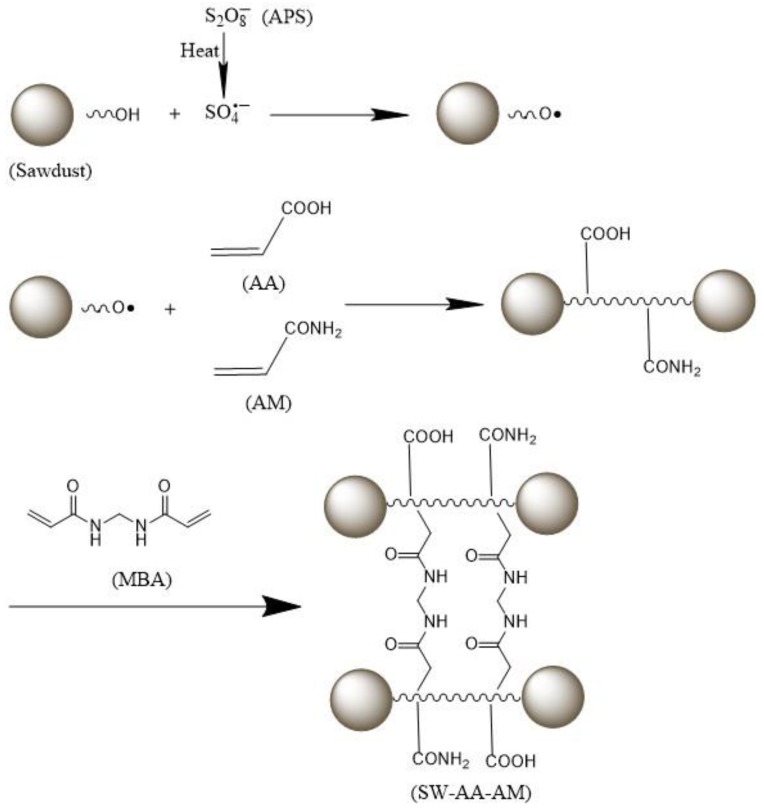
Mechanistic diagram of SW-AA-AM polymer formation.

The SW-AA-AM polymer was prepared by grafting the monomers (AM and AA) from the sawdust skeleton, by the initiator APS in the presence of the crosslinking agent MBA. The mechanism of SW-AA-AM polymer formation based on sawdust is shown in Figure 5. The initiator produces sulfate anion radicals under heating conditions [11]. The hydrogen atoms in the hydroxyl groups belonging to the sawdust are taken away by the radicals to become active sites, and acrylic acid and acrylamide are grafted at these positions to form polymer chains and start to grow. The SW-AA-AM polymer, having a network structure, is then formed by crosslinking the polymer chain with the crosslinking agent MBA.

### 3.3. Swelling Performance

To obtain the SW-AA-AM polymer with optimal swelling rate, six factors were studied, including the initiator content, the crosslinker content, the AA content, the AM content, the degree of neutralization of AA, and the reaction temperature of the experiment. Table 1 shows the formulation details used in polymer synthesis.

#### 3.3.1. Effect of MBA Content on the Swelling Rate

**Figure 6:**
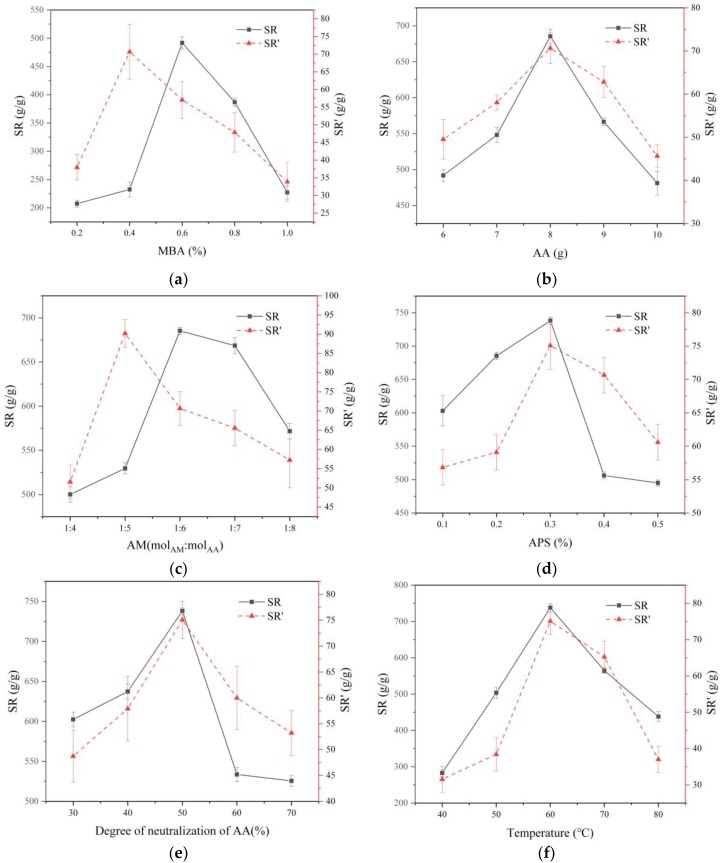
(**a**) Effect of the MBA content on the swelling rate. (**b**) Effect of the AA content on the swelling rate. (**c**) Effect of the AM content on the swelling rate. (**d**) Effect of the APS content on the swelling rate. (**e**) Effect of the neutralization of AA on the swelling rate. (**f**) Effect of the temperature on the swelling rate (continuous line represents SR; dotted line represents SR’. AA, acrylic acid; AM, acrylamide; MBA, *N,N’*-methylenebisacrylamide; APS, ammonium persulfate).

Figure 6a shows the effect of the MBA on the SAP swelling rate. The crosslinker content can influence the water absorption capacity of the SAP. The SR increased from 207.34 to 491.84 g/g with the MBA content changing from 0.2% to 0.6%. This is due to the poor crosslink density of the polymer at low crosslinker content, which does not lead to a stable network structure. The performance of the polymer in terms of water absorption and water retention are accordingly affected. The increase in the crosslinker causes an increase in the crosslinking site and density of the polymer, and the swelling rate of the polymer is further improved. However, with the increase of MBA content, the SR and SR’ show a decreasing trend. When the content of the crosslinker is too high, the voids in the network structure of the polymer would shrink, so that the swelling property of the polymer is inhibited.

#### 3.3.2. Effect of AA Content on the Swelling Rate

As shown in Figure 6b, the effect of AA amount on the SAP swelling rate was investigated. The swelling rate of SAP increased as the AA content increased from 6 to 8 g. It is mainly because the increase of AA dose promotes the collision probability between monomers, which is beneficial to the linear expansion of the polymer chain. It is conducive to the formation of network structure, so the polymer has more space to absorb and store moisture. When the amount of AA exceeds 8 g, the swelling rate of SAP showed an opposite trend. It is because of the self-polymerization caused by the excessive amount of AA, which increases the solubility of the polymer and results in a decrease in the swelling rate.

#### 3.3.3. Effect of AM Content on the Swelling Rate

The effect of AM:AA molar ratio on the SAP swelling rate is shown in Figure 6c. The results show that when the molar ratio of AM to AA is 1:6, the swelling rate of the polymer is as high as 685.36 g/g. It is the reason that the hydrophilicity of –COOH and –COONa in acrylic acid is superior to that of –CONH_2_ in acrylamide [50]. Moreover, the active groups between these two monomers may have a synergistic effect. The addition of AM increases the mechanical strength of the polymer so that the swollen polymer can maintain a stable shape. However, as the amount of AM increases, the hydrophobicity of the polymer becomes more pronounced, causes the swelling rate to further decrease.

#### 3.3.4. Effect of APS Content on the Swelling Rate

Figure 6d presents the effect of the APS on the SAP swelling rate. As the amount of APS increases from 0.1% to 0.3%, the swelling rate of SAP increases. When the amount of the APS is 0.3 mol %, the swelling rate of SAP reaches a maximum value. This is due to the fact that the rise in the initiator increases the concentration of free radicals in the solution, leading to more polymer graft sites and increasing the swelling rate of the SAP. However, with the further increase of the amount of the initiator, the swelling rate of SAP lowered. Too many free radicals will cause a chain termination reaction, so the graft chain of the polymer is shortened, and the spatial network structure will be difficult to form.

#### 3.3.5. Effect of the Neutralization of AA on the Swelling Rate

Figure 6e reveals the effect of the degree of neutralization of AA on the SAP swelling rate. The swelling rate of the polymer increases as the degree of AA neutralization increases from 30% to 50%. As the degree of AA neutralization further increases, the swelling rate of the polymer gradually decreases. Neutralization of AA with sodium hydroxide prevents the self-polymerization of highly active AA and the formation of dense polymers. At the same time, the hydrophilic group (–COONa) and the osmotic pressure of the polymer increase, which is favorable for the swelling rate of the polymer [51]. However, when the degree of neutralization of AA is higher than 50%, the activity of the AA monomer is decreased, and the ionic repulsive force in the network structure is lowered, leading to a decrease in the swelling rate of the polymer. However, when the degree of neutralization of AA is higher than 50%, the swelling rate of the polymer is lowered. It is caused by a decrease in the activity of the AA monomer and a decrease in the repulsive force between the ions.

#### 3.3.6. Effect of Temperature on the Swelling Rate

Figure 6f demonstrates the effect of reaction temperature on SAP water absorption. The results show that the swelling rate of the polymer first rises and then decreases as the temperature increases. The swelling rate of the polymer reached a maximum (738.12 g/g in distilled water and 90.18 g/g in 0.9 wt % NaCl solution) at 60 °C. The increase in temperature promotes the release and diffusion of free radicals, which is beneficial to the generation of reaction sites and the growth of chains, resulting in an increase in the swelling rate of the polymer [51]. However, when the temperature is too high, excessive free radicals are generated, which will accelerate the chain termination reaction to form a dense network structure, so the swelling rate of the polymer will decrease.

With the optimized synthesis conditions, the SR value increased from 207.34 to 738.12 g/g, while the SR’ value increased from 31.54 to 90.18 g/g. The swelling rate of the polymer in water is about seven times greater than that of the graft copolymer of xanthan gum-g-acrylamide-g-acrylic acid [39] and about two times that of the graft copolymer of protein-poly(acrylic acid-co-acrylamide) [52]. In addition, the swelling rate of the polymer in the NaCl 0.9% solution is much higher than that of the graft copolymers of chitosan-g-poly(acrylic acid)/attapulgite [16] and poly(acrylic acid-co-acrylamide)/sodium humate [15].

### 3.4. Water Retention Capacity

**Figure 7:**
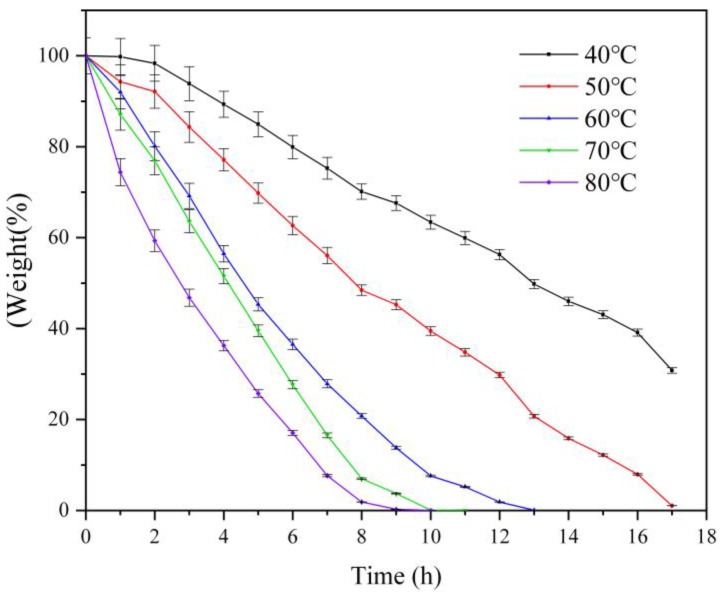
Water retention of SW-AA-AM polymer.

Figure 7 shows the water retention of SW-AA-AM at different temperatures. It can be observed from the image that the water content in the swollen polymer gradually decreases over time. The moisture in the polymer was exhausted after being dried at 80 °C for 10 h, while 63% of water was maintained at 40 °C. The moisture of the polymer remained at 30% after drying at 40 °C for 17 h. These phenomena can be explained by the interaction of hydrogen bonds and the existence of van der Waals forces between the polymer and water molecules.

### 3.5. Reusability

SAP with excellent reusability not only has high economic value, but also can relieve the burden of environmental pollution. Table 2 shows the results of the recycling test of SW-AA-AM. It can be seen that the SR value of the polymer decreases as the “swelling–drying” cycle increases. It is owing to the fact that the structure of the polymer is gradually destroyed [48]. After five cycles, the SR value of SW-AA-AM is still very high, reaching 327.36 g/g, and the R value is 0.44. This is because the continuous porous structure of the SW-AA-AM gives it excellent dimensional stability. It can be concluded that SW-AA-AM has excellent reusability and is a cost-effective polymer product with long service life and good application prospect.

### 3.6. Water Retention Performance in Soil or Coal

**Figure 8:**
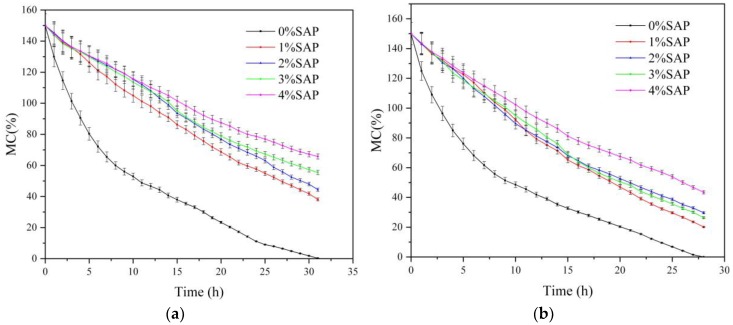
(**a**) Water retention of SW-AA-AM polymer in soil. (**b**) Water retention of SW-AA-AM polymer in coal.

The performance of the SW-AA-AM polymer was studied as a water retention agent for soil or coal. The samples (soil or coal) containing different amounts of SW-AA-AM polymer (0% SAP as the control) had an initial moisture content of 150%, as shown in Figure 8. In Figure 8a, the MC of the soil without SAP reduced vastly to 72% during the initial 6 h, and reached 0% after 31 h. The addition of SW-AA-AM polymer significantly reduces the rate of water volatilization in soil. The final moisture content of the 4% SAP-treated soil was 66%. At the same time, the soil moisture content containing only 1% SAP was still 38%. Similar results have also appeared in the coal test (Figure 8b). The interaction of hydrogen bonds and the existence of van der Waals forces between the polymer and water molecules can reduce the volatilization of water in the soil or coal [53].

**Figure 9:**
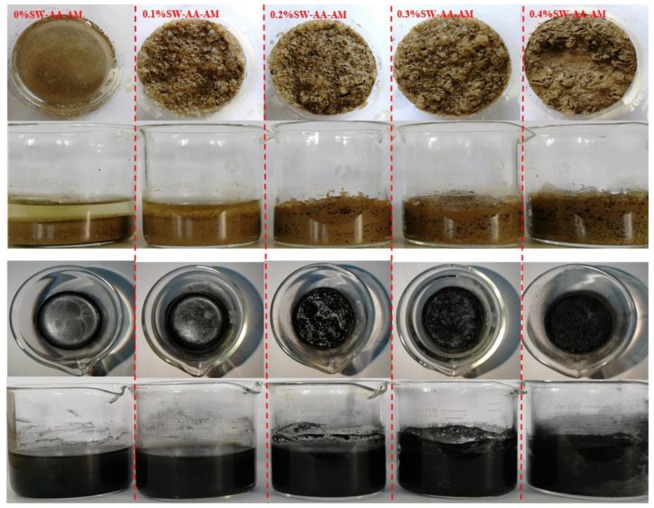
Water retention properties of SW-AA-AM polymer in soil and coal.

It can be clearly seen from Figure 9 that the SW-AA-AM polymer increases the porosity of the soil, indicating that the polymer improves the arrangement and combination of soil particles. This is attributed to the fact that the volume of the polymer increases after swelling, and the gap among the soil particles increases. It may be beneficial for the maintenance of the activity of soil microbes [54,55]. In addition, the saturated moisture of the samples increases as the content of polymer increases. This suggests that the presence of the polymer not only stores more water inside the sample to resist subsequent water volatilization, but also prevents the rapid loss of moisture on the surface of the sample. These experiments show that the synthesized SW-AA-AM polymer has great potential in promoting crop growth and dust suppression.

## 4. Conclusions

The SW-AA-AM superabsorbent polymer was prepared using sawdust without any chemical pretreatment. Sawdust serves as the framework structure and AA and AM as grafting monomers. The functional groups, crystallinity, and microscopic surface morphology of sawdust and the SW-g-AA-g-AM polymer were analyzed by FTIR, XRD, and SEM, respectively, which confirmed that the SW-AA-AM polymer was successfully prepared. The experimental results confirmed that the optimum swelling rate of the SW-AA-AM polymer can be attained under the following conditions: Crosslinker content, 0.6%; AA content, 8 g; the ratio of AM:AA, 1:6; initiator content, 0.3%; degree of neutralization of AA, 50%; reaction temperature, 60 °C. The SW-AA-AM polymer exhibits excellent swelling ability, thermal stability, and reusability. Furthermore, the SW-AA-AM polymer can be used as a water retention agent for soil or coal. The strategy of preparing superabsorbent polymers based on sawdust not only provides a new approach to the utilization of sawdust, but also brings a potential option for the preparation of cost-effective and environmentally-friendly industrial superabsorbent polymers.

## Figures and Tables

**Table 1:** The formulation details of SW-AA-AM polymers.

Group	MBA/AA (%)	AA (g)	AM:AA	APS/AA (%)	Neutralization Value (%)	Temperature (°C)
1	0.2	6	1:6	0.6	50	60
2	0.4	6	1:6	0.6	50	60
3	0.6	6	1:6	0.6	50	60
4	0.8	6	1:6	0.6	50	60
5	1.0	6	1:6	0.6	50	60
6	0.6	6	1:6	0.6	50	60
7	0.6	7	1:6	0.6	50	60
8	0.6	8	1:6	0.6	50	60
9	0.6	9	1:6	0.6	50	60
10	0.6	10	1:6	0.6	50	60
11	0.6	8	1:4	0.6	50	60
12	0.6	8	1:5	0.6	50	60
13	0.6	8	1:6	0.6	50	60
14	0.6	8	1:7	0.6	50	60
15	0.6	8	1:8	0.6	50	60
16	0.6	8	1:6	0.1	50	60
17	0.6	8	1:6	0.2	50	60
18	0.6	8	1:6	0.3	50	60
19	0.6	8	1:6	0.4	50	60
20	0.6	8	1:6	0.5	50	60
21	0.6	8	1:6	0.3	30	60
22	0.6	8	1:6	0.3	40	60
23	0.6	8	1:6	0.3	50	60
24	0.6	8	1:6	0.3	60	60
25	0.6	8	1:6	0.3	70	60
26	0.6	8	1:6	0.3	50	40
27	0.6	8	1:6	0.3	50	50
28	0.6	8	1:6	0.3	50	60
29	0.6	8	1:6	0.3	50	70
30	0.6	8	1:6	0.3	50	80

Note: SW-AA-AM, sawdust-graft-acrylic acid-graft-acrylamide; AA, acrylic acid; AM, acrylamide; MBA, N,N’-methylenebisacrylamide; APS, ammonium persulfate.

**Table 2:** The reusability of SW-AA-AM polymer.

Repeat Times	1	2	3	4	5
SR (g/g)	738.12	676.71	537.86	396.59	327.36
r	1	0.92	0.73	0.54	0.44

Note: SR, the swelling rate of the SW-AA-AM polymer.

## References

[1] Shen J., Cui C., Li J., Wang L. (2018). In Situ Synthesis of a Silver-Containing Superabsorbent Polymer via a Greener Method Based on Carboxymethyl Celluloses. Molecules.

[2] Shen J., Li B., Zhan X., Wang L. (2018). A one pot method for preparing an antibacterial superabsorbent hydrogel with a Semi-IPN structure based on tara gum and polyquaternium-7. Polymers.

[3] Olad A., Pourkhiyabi M., Gharekhani H., Doustdar F. (2018). Semi-IPN superabsorbent nanocomposite based on sodium alginate and montmorillonite: Reaction parameters and swelling characteristics. Carbohydr. Polym..

[4] Omidian H., Rocca J.G., Park K. (2005). Advances in superporous hydrogels. J. Control. Release.

[5] Zohuriaan-Mehr M.J., Omidian H., Doroudiani S., Kabiri K. (2010). Advances in non-hygienic applications of superabsorbent hydrogel materials. J. Mater. Sci..

[6] Luo M.T., Li H.L., Huang C., Zhang H.R., Xiong L., Chen X.F., Chen X. (2018). De Cellulose-based absorbent production from bacterial cellulose and acrylic acid: Synthesis and performance. Polymers.

[7] Yang S.T., Park Y.S. (2018). Release pattern of dexamethasone after administration through an implant-mediated drug delivery device with an active plunger of super absorbent polymer. Drug Deliv. Transl. Res..

[8] Yang L., Yang Y., Chen Z., Guo C., Li S. (2014). Influence of super absorbent polymer on soil water retention, seed germination and plant survivals for rocky slopes eco-engineering. Ecol. Eng..

[9] Alharbi K., Ghoneim A., Ebid A., El-Hamshary H., El-Newehy M.H. (2018). Controlled release of phosphorous fertilizer bound to carboxymethyl starch-g-polyacrylamide and maintaining a hydration level for the plant. Int. J. Biol. Macromol..

[10] Fahmy T.Y.A., Mobarak F. (2011). Green nanotechnology: A short cut to beneficiation of natural fibers. Int. J. Biol. Macromol..

[11] Liu X., Yang R., Xu M., Ma C., Li W., Yin Y., Huang Q., Wu Y., Li J., Liu S. (2018). Hydrothermal Synthesis of Cellulose Nanocrystal-Grafted-Acrylic Acid Aerogels with Superabsorbent Properties. Polymers.

[12] Farag A.M., Sokker H.H., Zayed E.M., Nour Eldien F.A., Abd Alrahman N.M. (2018). Removal of hazardous pollutants using bifunctional hydrogel obtained from modified starch by grafting copolymerization. Int. J. Biol. Macromol..

[13] Riyazi S., Kevern J.T., Mulheron M. (2017). Super absorbent polymers (SAPs) as physical air entrainment in cement mortars. Constr. Build. Mater..

[14] Shen D., Shi H., Tang X., Ji Y., Jiang G. (2016). Effect of internal curing with super absorbent polymers on residual stress development and stress relaxation in restrained concrete ring specimens. Constr. Build. Mater..

[15] Zhang J., Li A., Wang A. (2006). Synthesis and characterization of multifunctional poly(acrylic acid-co-acrylamide)/sodium humate superabsorbent composite. React. Funct. Polym..

[16] Zhang J., Wang Q., Wang A. (2007). Synthesis and characterization of chitosan-g-poly(acrylic acid)/attapulgite superabsorbent composites. Carbohydr. Polym..

[17] Song W., Xin J., Zhang J. (2017). One-pot synthesis of soy protein (SP)-poly(acrylic acid) (PAA) superabsorbent hydrogels via facile preparation of SP macromonomer. Ind. Crops Prod..

[18] Lee J., Park S., Roh H.G., Oh S., Kim S., Kim M., Kim D., Park J. (2018). Preparation and characterization of superabsorbent polymers based on starch aldehydes and carboxymethyl cellulose. Polymers.

[19] Razali M.A.A., Ismail H., Ariffin A. (2015). Graft copolymerization of polyDADMAC to cassava starch: Evaluation of process variables via central composite design. Ind. Crops Prod..

[20] Álvarez-Castillo E., Del Toro A., Aguilar J.M., Guerrero A., Bengoechea C. (2018). Optimization of a thermal process for the production of superabsorbent materials based on a soy protein isolate. Ind. Crops Prod..

[21] Xie L., Liu M., Ni B., Zhang X., Wang Y. (2011). Slow-release nitrogen and boron fertilizer from a functional superabsorbent formulation based on wheat straw and attapulgite. Chem. Eng. J..

[22] Xie L., Liu M., Ni B., Wang Y. (2012). New environment-friendly use of wheat straw in slow-release fertilizer formulations with the function of superabsorbent. Ind. Eng. Chem. Res..

[23] Cheng W.M., Hu X.M., Wang D.M., Liu G.H. (2015). Preparation and characteristics of corn straw-Co-AMPS-Co-AA superabsorbent hydrogel. Polymers.

[24] Ma Z., Li Q., Yue Q., Gao B., Xu X., Zhong Q. (2011). Synthesis and characterization of a novel super-absorbent based on wheat straw. Bioresour. Technol..

[25] Fang J.M., Sun R.C., Tomkinson J. (2000). Isolation and characterization of hemicelluloses and cellulose from rye straw by alkaline peroxide extraction. Cellulose.

[26] Wan T., Huang R., Zhao Q., Xiong L., Qin L., Tan X., Cai G. (2013). Synthesis of wheat straw composite superabsorbent. J. Appl. Polym. Sci..

[27] Jin S., Chen J., Mao J., Yue G., Han Y., Yu X. (2017). A novel superabsorbent from raw corn straw and poly(acrylic acid). Polym. Compos..

[28] Dai D., Fan M. (2015). Preparation of bio-composite from wood sawdust and gypsum. Ind. Crops Prod..

[29] Jung C.H., Choi J.H., Lim Y.M., Jeun J.P., Kang P.H., Nho Y.C. (2006). Preparation and characterization of polypropylene nanocomposites containing polystyrene-grafted alumina nanoparticles. J. Ind. Eng. Chem..

[30] Rani G.U., Mishra S., Pathak G., Jha U., Sen G. (2013). Synthesis and applications of poly(2-hydroxyethylmethacrylate) grafted agar: A microwave based approach. Int. J. Biol. Macromol..

[31] Li Y.N., Sun Y., Deng X.H., Yang Q., Bai Z.Y., Xu Z. (2006). Bin Graft polymerization of acrylic acid onto polyphenylene sulfide nonwoven initiated by low temperature plasma. J. Appl. Polym. Sci..

[32] Lee J.Y., Park C.Y., Moon S.Y., Choi J.H., Chang B.J., Kim J.H. (2019). Surface-attached brush-type CO_2_-philic poly(PEGMA)/PSf composite membranes by UV/ozone-induced graft polymerization: Fabrication, characterization, and gas separation properties. J. Memb. Sci..

[33] Zhuo J., Sun G. (2014). Light-induced surface graft polymerizations initiated by an anthraquinone dye on cotton fibers. Carbohydr. Polym..

[34] Fang S., Wang G., Li P., Xing R., Liu S., Qin Y., Yu H., Chen X., Li K. (2018). Synthesis of chitosan derivative graft acrylic acid superabsorbent polymers and its application as water retaining agent. Int. J. Biol. Macromol..

[35] Wang K., Dong Y., Yan Y., Zhang W., Qi C., Han C., Li J., Zhang S. (2017). Highly hydrophobic and self-cleaning bulk wood prepared by grafting long-chain alkyl onto wood cell walls. Wood Sci. Technol..

[36] Qiu H., Yang S., Han Y., Shen X., Fan D., Li G., Chu F. (2018). Improvement of the Performance of Plantation Wood by Grafting Water-Soluble Vinyl Monomers onto Cell Walls. ACS Sustain. Chem. Eng..

[37] Cabane E., Keplinger T., Merk V., Hass P., Burgert I. (2014). Renewable and functional wood materials by grafting polymerization within cell walls. ChemSusChem.

[38] Suo A.L., Qian J.M., Yao Y., Zhang W.G. (2007). Synthesis and properties of carboxymethyl cellulose-graft-poly (acrylic acid-co-acrylamide) as a novel cellulose-based superabsorbent. J. Appl. Polym. Sci..

[39] Zheng M., Lian F., Zhu Y., Zhang Y., Liu B., Zhang L., Zheng B. (2019). pH-responsive poly (xanthan gum-g-acrylamide-g-acrylic acid) hydrogel: Preparation, characterization, and application. Carbohydr. Polym..

[40] Liu Z., Miao Y., Wang Z., Yin G. (2009). Synthesis and characterization of a novel super-absorbent based on chemically modified pulverized wheat straw and acrylic acid. Carbohydr. Polym..

[41] Wan T., Huang R., Xiong L., Zhao Q., Luo L., Zhang H., Cai G. (2014). Swelling behaviors and gel strength studies of wheat straw-composite superabsorbent. J. Compos. Mater..

[42] Li J. (2009). Wood Spectroscopy.

[43] Baki M., Abedi-Koupai J. (2018). Preparation and characterization of a superabsorbent slow-release fertilizer with sodium alginate and biochar. J. Appl. Polym. Sci..

[44] Fang S., Wang G., Xing R., Chen X., Liu S., Qin Y., Li K., Wang X., Li R., Li P. (2019). Synthesis of superabsorbent polymers based on chitosan derivative graft acrylic acid-co-acrylamide and its property testing. Int. J. Biol. Macromol..

[45] Bala R., Mondal M.K. (2018). Exhaustive characterization on chemical and thermal treatment of sawdust for improved biogas production. Biomass Convers. Biorefinery.

[46] Zhang Q., Huang H., Han H., Qiu Z., Achal V. (2017). Stimulatory effect of in-situ detoxification on bioethanol production by rice straw. Energy.

[47] Phitsuwan P., Sakka K., Ratanakhanokchai K. (2016). Structural changes and enzymatic response of Napier grass (Pennisetum purpureum) stem induced by alkaline pretreatment. Bioresour. Technol..

[48] Peppas N.A., Khare A.R. (1993). Preparation, structure and diffusional behavior of hydrogels in controlled release. Adv. Drug Deliv. Rev..

[49] Liu X., Luan S., Li W. (2019). Utilization of waste hemicelluloses lye for superabsorbent hydrogel synthesis. Int. J. Biol. Macromol..

[50] Wu L., Liu M. (2007). Slow-release potassium silicate fertilizer with the function of superabsorbent and water retention. Ind. Eng. Chem. Res..

[51] Pourjavadi A., Samadi M., Ghasemzadeh H. (2008). Fast-swelling superabsorbent hydrogels from poly(2-hydroxy ethyl acrylate-co-sodium acrylate) grafted on starch. Starch/Staerke.

[52] Zhang B., Cui Y., Yin G., Li X., Liao L., Cao X. (2011). Synthesis and swelling properties of protein-poly(acrylic acid-co-acrylamide) superabsorbent composite. Polym. Compos..

[53] Bao Q., Nie W., Liu C., Liu Y., Zhang H., Wang H., Jin H. (2019). Preparation and characterization of a binary-graft-based, water-absorbing dust suppressant for coal transportation. J. Appl. Polym. Sci..

[54] Yang W., Guo S., Li P., Song R., Yu J. (2019). Foliar antitranspirant and soil superabsorbent hydrogel affect photosynthetic gas exchange and water use efficiency of maize grown under low rainfall conditions. J. Sci. Food Agric..

[55] Li X., He J.Z., Liu Y.R., Zheng Y.M. (2013). Effects of super absorbent polymers on soil microbial properties and Chinese cabbage (Brassica chinensis) growth. J. Soils Sediments.

